# Oral Aminoacids Supplementation Improves Corneal Reinnervation After Photorefractive Keratectomy: A Confocal-Based Investigation

**DOI:** 10.3389/fphar.2021.680734

**Published:** 2021-07-26

**Authors:** Anna M Roszkowska, Dario Rusciano, Leandro Inferrera, Alice Antonella Severo, Pasquale Aragona

**Affiliations:** ^1^ Ophthalmology Clinic, Department of Biomedical Sciences, University of Messina, Messina, Italy; ^2^ Sooft Italia SpA Research Center, Catania, Italy

**Keywords:** aminoacids, PRK, cornea, corneal nerves, nerve growth, confocal microscopy

## Abstract

Aim of this retrospective study was to estimate the effect of oral supplementation with amino acids (AA) on corneal nerves regrowth after excimer laser refractive surgery with photorefractive keratectomy (PRK). Based on the pre and post-surgical treatment received, 40 patients with 12 months of follow-up were distributed in two groups: 20 patients had received oral AA supplementation 7 days before and 30 days after PRK, and 20 patients without AA supplementation, as untreated reference control. All patients followed the same standard post-operative topical therapy consisting of an association of antibiotic and steroid plus sodium hyaluronate during the first week, then steroid alone progressively decreasing during 30 days and sodium hyaluronate for the following 3 months. *In vivo* corneal confocal microscopy was used to evaluate the presence of sub-basal corneal nerve fibers during 12 months after PRK. Results have shown that sub-basal nerves regenerated significantly faster (*p* <0.05), and nerve fibers density was significantly higher (*p* <0.05) with a more regular pattern in the eyes of AA treated patients with respect to the untreated control group. Therefore, our data indicate that oral supplementation with AA improved significantly corneal nerve restoration after PRK and could thus be considered as an additional treatment during corneal surgical procedures.

## Introduction

Photorefractive keratectomy (PRK) is a common excimer laser procedure still widely used to correct refractive errors. PRK is a surface ablation procedure, in which the corneal epithelium is removed to permit the laser to reshape the anterior corneal stroma. The depth of ablation varies in relation to the refractive error, therefore different amounts of the sub-basal nerve plexus (SBNP) fibers are injured in relation to the ablation zone (Erie, 2003; Tomás-Juan et al., 2015; Labetoulle et al., 2019). While the epithelium heals within 3–5 days (Erie, 2003), the complete regeneration of the SBNP nerves occurs during several months (Erie, 2003). The interruption of the nerve reflex between the cornea and the lacrimal glands interferes with normal lacrimation, so that the ocular surface may be altered by insufficient moistening, thus triggering eye dryness and patient’s discomfort (Labetoulle et al., 2019). Additionally, the surgical procedure activates inflammation and keratocytes transformation to fibroblasts and myofibroblasts that produce normally absent type IV collagen and disorganized extracellular matrix, that together to the abnormal arrangement pattern of collagen type I and III results clinically in corneal haze (Tomás-Juan et al., 2015). Therefore, it is critical that wound repair (involving epithelium, stroma and nerve fibers) occurs reasonably fast and definitely well, to reduce patient’s discomfort and promote corneal healing thus allowing a fast and good recovery of vision.

Cell metabolism is tightly dependent on the availability of aminoacids (AA), which are necessary to build new macromolecules used in cell and extracellular matrix architecture. Moreover, AA may also have different functions, specifically linked to their molecular structure and interaction with cell regulatory mechanisms (Rusciano et al., 2016). The process of wound healing poses a serious challenge to cell metabolism, and in case of AA deficiency the whole process could be hampered, also impairing the immune defense, and finally exposing the tissue to an increased risk of infection (Herndon and Tompkins, 2004; Chandrasekaran et al., 2017). Some AAs are known to facilitate the process of wound healing (Stechmiller, 2010). Arginine triggers nitric oxide (NO) synthesis, thus stimulating collagen synthesis, antimicrobial activity, and blood flow (Alexander and Supp, 2014). Glutamine is an energy source and induces the expression of heat shock proteins, thus protecting the tissue from inflammation damage (Chow and Barbul, 2014). Leucine is normally metabolized into hydroxy-methylbutyrate (HMB), an active molecule blunting proteolysis, stimulating protein synthesis, decreasing apoptosis and increasing cell proliferation (Sipahi et al., 2013). An experimental model system evaluated wound healing in a skin full-thickness excisional model in rats receiving diets with a different content of essential AA. Results have shown that wound repair was accelerated, and inflammation reduced, in rats fed with an increased amount of essential AA (Corsetti et al., 2017). Oral AA supplementation has shown to help wound recovery in refractory patients after PRK (Vinciguerra et al., 2002) or cataract (Torres Munoz et al., 2003) surgery. Treatment with topical AA given as eye drops resulted in a better improvement of signs and symptoms in patients affected by dry eye (Sacchetti et al., 2012; Aragona et al., 2013).

Based on these premises, in this preliminary study we aimed to evaluate the regeneration of the sub-basal corneal nerve fibers in patients treated with standard post-surgical therapy who received an additional treatment with oral AA.

## Materials and Methods

This retrospective study included clinical and instrumental data from 40 eyes of 40 patients selected among those who were treated with PRK for myopia or composed myopic astigmatism and completed a follow-up of at least 12 months. Subjects were chosen from the database of the Cornea and Refractive Surgery section of Messina University Hospital Ophthalmology Unit. Inclusion criteria were: age between 20 and 40 years; spherical equivalent between −2.75 and −7 diopters with ablation depth between 45 and 100 microns; available follow up of 12 months. Among these subjects, all suitable for PRK, 20 were chosen who had received AA supplementation when the specific product was available, and 20 (as control reference group) who did not receive AA because treated in a previous period when the food supplement was not available.

The PRK surgical procedure followed the same protocol for all patients. Oxybuprocaine hydrochloride anesthetic drops (Novesina, Thea Farma, Italy) were instilled before the surgical treatment, and the epithelium was removed with a blunt spatula in an area of 9 mm diameter, after 20% alcohol delamination for 20 s. PRK was performed with a Mel-70 G-Scan excimer laser (Carl Zeiss, Jena, Germany) provided with flying spot with gaussian profile with a diameter of 1.8 mm. The ablation zone was 7 mm with 1.8 mm of transition. After treatment, a soft therapeutic contact lens was applied for 5 days and removed when the epithelium healed.

All patients followed the same standard post-operative therapy used in our center, consisting for the first 5 days of eye drops with dexamethasone 0.1% and tobramycin 0.3% (Tobradex, Alcon, Italy) one drop four q.i.d. plus sodium hyaluronate (Blu Yal, Sooft Italia) one drop four q.i.d. for 6 days until healing of the epithelium; then fluorometholone 0.2% unit dose eye drops (Flumetol, Thea Farma, Italy) one drop for 4 times q.i.d. for 10 days, then 3 times daily for 20 days and twice daily for 10 days plus sodium hyaluronate (Blu Yal, Sooft Italia) one drop four q.i.d. for the following 2 months.

The oral supplementation treatment with AA consisted of one tablet containing an AA mix (Aminoftal® Sooft Italia SpA: Table 1) three times daily for 7 days before and 30 days after PRK surgery. The files of all subjects in the treatment group reported completion of the prescribed AA administration, with no one complaining of disturbs, intolerance or adverse effects related to the AA supplementation received.

**TABLE 1:** L-aminoacid content per tablet (* denotes an essential AA).

Aminoacid	Amount (mg)
Leucine*	250
Lysine*	130
Isoleucine*	125
Valine*	125
Threonine*	70
Cystine	30
Histidine*	30
Phenylalanine*	20
Methionine*	10
Tyrosine	6
Tryptophan*	4

To assess their eligibility for the PRK procedure all patients were subjected to a complete ophthalmological examination with visual acuity (VA) assessment, refraction, slit lamp evaluation, tonometry and fundus. The instrumental evaluation comprised corneal topography, tomography, corneal thickness, Schirmer test, TBUT and *in vivo* corneal confocal microscopy (IVCM). VA, refraction, corneal topography, pachymetry and IVCM were reassessed at 1–3–6 and 12 months after PRK.

IVCM was performed with the Confoscan 4 (Nidek Technologies, Vigonza, Italy), equipped with the Z-Ring to provide central image acquisition. The presence of nerve fibers of SBNP was detected at each time point and three fields for each eye were examined by the same experienced operator on 0.159 mm^2^ frames. Sub-basal nerve plexus (SBNP) fibers were analyzed by the ImageJ/Neuron-J plug in (National Institute of Health, Bethesda, United States), a semiautomated software for nerve tracing and analysis showing a high interobserver repeatability (Cottrell et al., 2014; Dehghani et al., 2014).

The nerve fibers density (NFD) was calculated in μm/mm^2^ and an average value was considered for calculations.

The number of subjects enrolled in the present study (*n* = 40: 20 controls and 20 AA-treated) was based on statistical power calculation made with G Power software version 3.1 setting the power to 0.80, α to 0.05 and considering a large effect size (on the basis of previous studies, showing how slow is nerve regeneration after PRK (Erie, 2003). Statistical analysis was performed with Graph Pad Prism (version 8). Fisher’s exact test was used for non-parametric data (presence of nerve fibers), whereas unpaired Student’s T-test was used to analyze the parametric data normally distributed (age, refractive error, ablation depth, NFD).

The study was approved by the Ethical Committee of the University Hospital of Messina (N. 65/18).

## Results

The demographic characteristics of patients included in this study are represented in Table 2. Both groups were homogeneous for sex, age, refractive error and depth of ablation (*p* > 0.05). Their spherical equivalent refraction was comprised between −5 and −6 diopters, and the mean ablation depth was 68.71 ± 19.7 mm in the AA group and 67.25 ± 21.5 mm in the control group (Table 2).

**TABLE 2:** Characteristics of enrolled patients.

Group	Males	Females	AGE (years) ± SD	Diopters ± SD	Ablation depth (mm) ± SD
**AA**	9	11	30.42 ± 4.45	-5.40 ± 2.2	68.71 ± 19.7
**Control**	8	12	31.08 ± 4.42	-5.92 ± 1.81	67.25 ± 21.5

**FIGURE 1:**
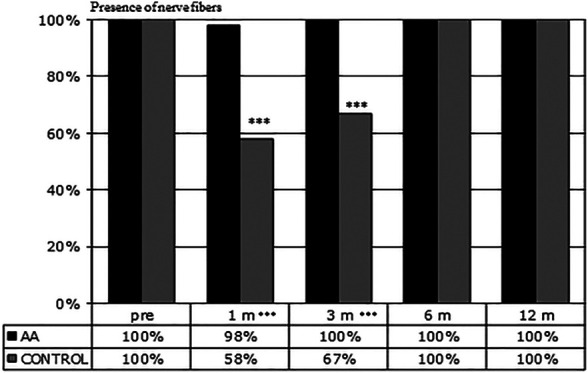
Presence of nerve fibers during follow-up. Evaluation of the number of fields (3 fields/patient) presenting nerve fibers at the indicated time points. At 1 and 3 months corneal nerve regeneration resulted to be significantly faster in patients treated with AA than in the control group (***p < 0.001 by Fisher’s exact test).

Figure 1 shows the percentage of eyes in which nerve fibers could be detected in selected confocal microscopy fields. After one-month, nerve fibers were present in 98% of eyes in the AA group and 58% in the control group (*p* <0.001). A significant difference in the amount of SBNP fibers was still present at 3 months (100 vs 67% *p* < 0.001), whereas at 6 and 12 months in all eyes SBNP fibers could be detected. These results indicate that corneal nerve regeneration is significantly faster in patients who received oral AA supplementation than in the control group not treated with AA.

**FIGURE 2:**
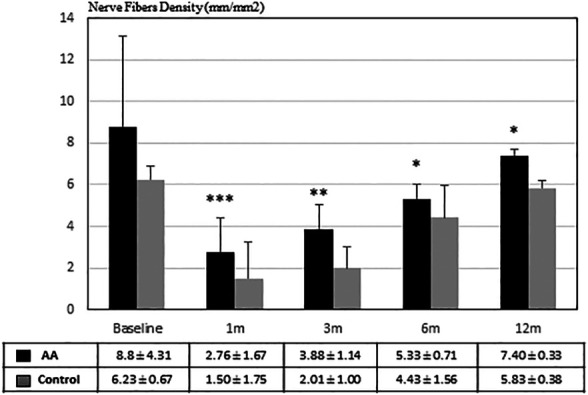
Nerve Fibers Density (μm/mm^2^) during follow-up. Nerve regeneration resulted to be significantly faster in patients treated with AA than in the control group (*p <0.05; **p <0.01; ***p < 0.001 by Student’s t-test).

Figure 2 shows SBNP nerve fibers density (NFD) calculated on confocal images in both groups during the follow-up between 1 month and 1 year. It is evident that NFD increased faster in patients treated with AA than in the control group. In fact, at each time point after PRK, the NFD was significantly higher in the eyes of AA treated patients than in control eyes (*p* < 0.001 at 1 month; *p* < 0.01 at 3 months; *p* < 0.05 at 6 and 12 months).

**FIGURE 3:**
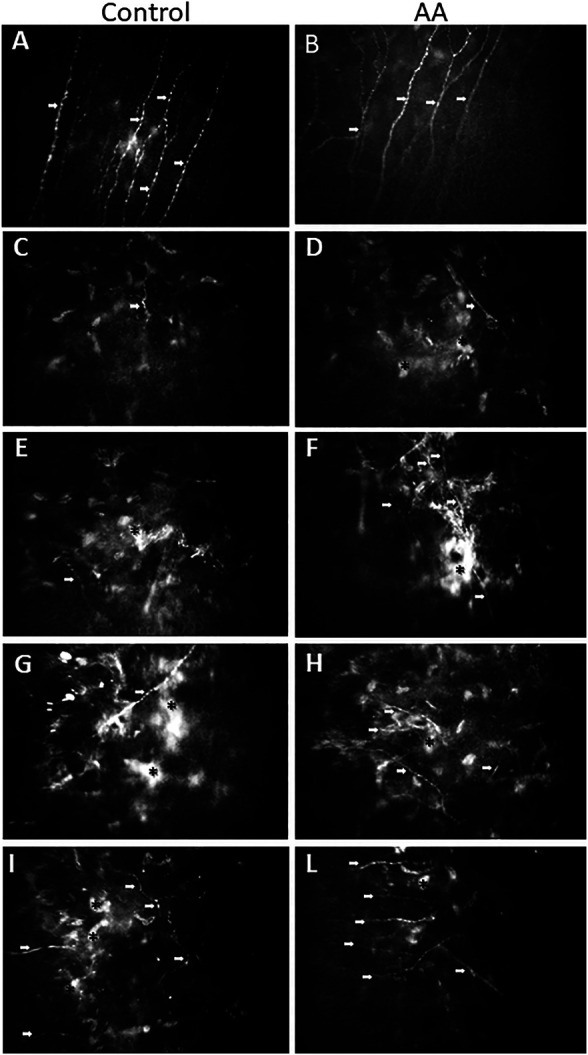
Representative confocal images taken where the SBNP fibers best appear on focus. **(A)**: image taken from a control patient before PRK; **(B)**: image taken from an AA-treated patient before PRK. Both pictures show the normal path of SBNP fibers, which appear with a regular, almost parallel pattern. Representative confocal images of SBNP nerve fibers were taken at different times after PRK of the control patient (left column: treated for −3.50 D of SE with ablation of 67 microns) and of the AA-treated patient (right column: treated for −4.50 D of SE with ablation of 70 microns). After 1 month from PRK there is an initial presence of nerve fibers (among activated, hyper-reflective stromal cells) in the AA group **(D)**, while in the control group **(C)** no nerves or sporadic fibers could be detected. After 3 months from PRK an increased number of SBNP fibers is apparent in the AA group **(F)**, while in the control group **(E)** some nerve fibers start to be detectable. After 6 months from PRK all examined eyes exhibit SBNP fibers, but the density is significantly higher in the AA group **(H)** than in control eyes **(G)**. After 12 months from PRK the nerve fiber density is higher in the AA group, also showing a more regular linear pattern (L) than in control eyes of patients not treated with AA **(I)**. White arrows indicate growing fibers; asterisks indicate activated, hyper-reflective stromal cells.

Figure 3 shows the evolution as seen at confocal microscopy of SBNP fibers repopulation in the subepithelial anterior corneal stroma after PRK at progressive times up until one year. Figures 3A,B illustrate the normal sub basal nerve fibers pattern with a regular almost parallel path at baseline evaluation, just before ablation. Pictures on the left column (Figures 3C–E) are representative of untreated control patients: Pictures on the right column (Figures 3D–F) are representative of AA-treated patients. Figures 3C,D illustrates the appearance of subepithelial corneal stroma at 4 weeks after the intervention in subjects treated with oral aminoacids (D) or untreated controls (C). Some short and bundled nerve fibers start to be apparent in AA-treated patients among activated, hyper-reflective stromal cells, while no such evidence is present in control pictures, showing only the activated stromal cells. The situation at 3 months after PRK is shown in Figures 3E,F: an increasing number of sub-basal nerve plexus fibers is evident in AA-treated patients (F), while in controls nerve fibers start to make their appearance (E). At 6 months (Figures 3G,H) all eyes show the presence of growing nerve fibers, however with a higher density in the eyes of patients coming from AA (H) treatment group with respect to controls (G). A similar finding is still visible after 12 months from the intervention (Figures 3I–L), with confocal pictures of eyes of patients treated with AA (L) showing a higher density of nerve fibers, with a more regular pattern, while in control eyes nerve fibers continue to show an irregular pattern, among still highly hyperreflective stromal cells (I).

## Discussion

The results of this confocal-based retrospective study address for the first time the basic role of AA supplementation on corneal peripheral nerve regeneration after PRK. Our data show that a diet enriched in essential AA may enhance the regeneration of SBNP fibers lesioned by the PRK intervention, so that their density and pathway through the regenerating stroma is improved, and healing is achieved in shorter times with respect to patients in which the AA supplement was not given.

With 16,000 nerve terminations/mm^3^, the cornea is the most densely innervated tissue of the human body (Guthoff et al., 2005). Therefore, PRK ablation, destroying SBNP fibers in the central cornea, makes the procedure very painful, since the remaining nerve endings after laser ablation are exposed at the surface until the epithelium regenerates and covers the surgical wound (Mohan et al., 2003). While epithelium regrowth occurs within few days (Olivieri et al., 2018), stromal remodeling and nerve regeneration are much slower events (Tomás-Juan et al., 2015). Even though new nerve fibers may appear from the ablation area within the first weeks, only half reinnervation is completed at 6 months, returning to pre-operative density only after 2 years (Erie et al., 2005). Such prolonged corneal nerve density reduction may lead to ocular surface discomfort and dry eye disease with different severity grade (Dohlman et al., 2016). Occasionally, more severe consequences may happen, such as hypoesthesia, neurotrophic ulcers, or chronic inflammation (Chao et al., 2014). Aberrant reinnervation may also occur and lead to allodynia or keratoneuralgia (Hamrah et al., 2017). Therefore, the timing and the quality of reinnervation after PRK are critical, so to rescue the correct corneal physiological behavior of the ocular surface functional unit (Stern et al., 2004). Keratocyte repopulation and healing of the ablated corneal stroma supply growth factors and biological cues guiding the correct regeneration of lesioned nerve endings. In fact, further to stromal ablation, the surviving keratocytes in corneal stroma are activated to stromal fibroblasts (SFs), which secrete neurotrophic and inflammatory factors regulating neurogenesis and wound healing (Yam et al., 2017). The regenerating corneal epithelium is also a source of neurotrophic growth factors, such as NGF and GDNF, also contributing towards nerve regrowth (Di et al., 2017).

Several different topical treatments with eye drops have been tried to improve corneal nerve regeneration after keratectomy. Murine NGF purified from the submaxillary gland was used in a corneal flap rabbit model of LASIK and shown to accelerate the recovery of corneal sensitivity as measured by esthesiometry (Joo et al., 2004) and promote nerve regeneration as visualized by confocal microscopy (Ma et al., 2014). With the same rabbit model, a bioactive N-terminal peptide derived from the pituitary adenylate cyclase–activating polypeptide (PACAP27) has been shown to be able to promote neurite outgrowth as followed by histological analysis (Fukiage et al., 2007). Topical applications of PEDF in association with DHA also improved the recovery of sensitivity in rabbit corneas subjected to PRK (Cortina et al., 2012). In rodent models of corneal epithelial debridement, topical administration as eye drops of vitamin B12 and taurine, together with sodium hyaluronate, enhanced both re-epithelization and re-innervation (Romano et al., 2014). Finally, Ginkgo Biloba/hyaluronic acid (GB/HA) eye drops were given to a group of 15 patients (30 eyes) after PRK surgery and compared to hyaluronic acid alone. Confocal microscopy analysis performed at 1–3–6–9–12 months showed a clear advantage in both quantitative and qualitative effects in the GB/HA group (Bisantis, 2006). More recently, systemic administration of a secondary metabolite (epothilone B) produced by the *mycobacterium* Sorangium cellulosum, which is a microtubule stabilizing agent, has been found to lead to a favorable pharmacodynamics in the cornea and corneal nerves, and to speed up corneal reinnervation after epithelial debridement in mice (Wang et al., 2018).

However, whichever treatment is given to stimulate nerve regeneration, the necessary requirement in all instances is that enough “building” material is available to nerve cells and their neighboring cells to elongate their nerve endings in a permissive environment, in which stromal cells produce an extracellular matrix (ECM) favoring the whole process (Gonzalez-Perez et al., 2013). In fact, all these activities require new protein (structural and enzymatic) synthesis to allow and speed up the series of events leading to the restoration of the perturbed homeostasis of the tissue. This is also evident in our study, in which the relative amount of activated (hyper-reflective) stromal cells remains higher in control patients than in AA-treated patients (Figure 3), especially visible after 12 months (Figures 3I–L).

The availability of AA, and more specifically of the essential AA, those that must be taken through the diet, can be a limiting step in this process. Like what happens with muscle exercise, where mechanical stimulation results more effective in inducing growth of the muscle fibers in presence of an increased concentration of essential AA in blood (Church et al., 2020), also for nerve regeneration a similar situation may occur. This has been clearly shown in the goldfish model of retinal ganglion cells regeneration, in which transected ganglion cells demonstrate a marked increase in protein biosynthesis as their axons regrow into a primary target tissue (Giulian, 1984) and in which extracellular amino acids strongly contribute to the composition of the immediate precursor pool for protein synthesis in regenerating cells (Whitnall and Grafstein, 1981). A recent review on the effects of nutrition-related factors on peripheral nerve injuries highlighted the role of omega fatty acids, vitamins, antioxidants and proteins rich in essential AA in preserving nerve function and health, and in the recovery of injured tissue (Yildiran et al., 2020). In this respect, the bioavailability of free AA taken as food supplement is expected to be higher than that deriving from the metabolism of ingested food proteins, that require a complex enzymatic pathway and absorption process, and depends on the particular diet habits of the subject (Schmidt et al., 2016).

Moreover, AA may also have other functions beside the building of proteins, such as antioxidants or participate in the chain of neurotransmission. For instance, in a rat model of facial nerve crush injury systemic administration of n‐acetylcysteine (a potent antioxidant) favored nerve recovery as shown by improved functional and electromyography outcomes (Rivera et al., 2017). AA can be utilized to synthesize both lipids and glucose. Increased intake of essential AA may increase laboratory animals’ lifespan through the activation of Sirt-1 dependent mitochondrial biogenesis (D’Antona et al., 2010). Clinical studies in humans have addressed the role of essential AA in enhancing protein synthesis independently from age, and improving muscle catabolism in the elderly during prolonged bed rest (Cuthbertson et al., 2005; Ferrando et al., 2010). Finally, a metanalysis of clinical studies on patients with painful peripheral neuropathy showed that a food supplement of acetyl-L-carnitine (a precursor of neurotransmitters derived from the AA lysine and methionine) exerted several beneficial effects on nerve conduction parameters and nerve fiber regeneration, generally improving patients’ condition (Di Stefano et al., 2019). All these effects may have both a direct and an indirect relevance because the healing of a tissue or an organ in the body depends on the state of the tissue or the organ by itself, but also from the general health condition of the body in which it resides. Therefore, since an adequate intake of essential AA can also improve the general health condition of the patient, this effect can reverberate on the healing properties of different organs, eye included (Pache and Flammer, 2006). Consistent with these premises, our results show that the intake of supplemental AA has a higher impact during the first 3 months, improving the initial stages of SBNP repopulation: in fact, a faster and wider repopulation of SBNP fibers is evident at 1 and 3 months in AA-treated subjects (Figure 1), while the statistical significance of NFD comparative data between treated and untreated patients becomes progressively lower at 6 and 12 months, however still pointing at some advantage for AA treated subjects (Figure 2). A similar treatment with the same AA food supplement was already reported in a previous study (Vinciguerra et al., 2002), in which patients with chronic epithelial defects, or with delayed re-epithelization times after PRK showed an improved healing response after oral AA treatment. Similarly, supplementation with the same AA mixture improved corneal stromal regeneration after cataract surgery (Torres Munoz et al., 2003). This finding is relevant also in our study, because keratocyte and stromal healing is critical for correct nerve growth (Gonzalez-Perez et al., 2013), thus suggesting that AA may contribute both a direct (on nerve cells themselves) and an indirect (through keratocytes and the stroma) effect on SBNP restoration.

A limitation of these observations is the small number of patients under scrutiny; to be a retrospective study (therefore, no clinical data on eye dryness were available, and no pharmacokinetics could be programmed) conducted in a single center; and to be the first of its kind. Nonetheless, data obtained are very promising, and grant a prosecution of the study, in a prospective way and on a higher number of patients.

## Conclusion

In this study we have clearly shown throughout 12 months of confocal microscopy observations that SBNP fibers lesioned during PRK grow faster and better when patients take a supplement of essential AA. Data presented in this study corroborate the function of exogenous AA in corneal nerve regeneration, in line with previous studies showing the role of supplemental AA given either as food supplement (Vinciguerra et al., 2002; Torres Munoz et al., 2003) or as topical eye drops (Sacchetti et al., 2012; Aragona et al., 2013) in the healing of the cornea and corneal nerves after surgical interventions or during the course of dry eye. It is likely that the effect of AA supplementation by improving both epithelial and stromal healing, and by providing a favorable growth milieu to regenerating nerve cells, finally results in a faster and better reinnervation of the lesioned cornea, most evident during the first 3 months post-surgery.

In conclusion, our results support the use of food supplements enriched with essential AA, or at least a diet rich in AA content, before and after PRK surgery, or in case of any surgical intervention that involves tissue and nerve regeneration.

## Data Availability

The data analyzed in this study is subject to the following licenses/restrictions: Data on file at the Ophthalmology Dpt. Of the University of Messina (Italy). Requests to access these datasets should be directed to anna.roszkowska@unime.it
